# Not all meniscal repair failures are equal: A comparison between early and late failure risk factors

**DOI:** 10.1002/jeo2.70856

**Published:** 2026-07-20

**Authors:** Jacob W. McDevitt, Thomas J. Policicchio, Anoop Sunkara, Cade F. Bennett, Michael O. Sohn, Jake A. Morales, Vehniah K. Tjong

**Affiliations:** ^1^ Department of Orthopaedic Surgery Northwestern University Feinberg School of Medicine Chicago Illinois USA

**Keywords:** cause‐specific hazard regression, knee osteoarthritis, meniscal repair, reoperation, repair failure, risk stratification

## Abstract

**Purpose:**

Meniscal repair failure is a significant clinical concern, but existing literature treats it as a single outcome without differentiating timing. Early and late failures may represent mechanistically distinct phenomena; identifying their differential predictors could improve risk stratification and postoperative monitoring.

**Methods:**

A retrospective cohort study used a national database to identify patients undergoing arthroscopic meniscal repair, including both isolated repairs and repairs performed with concomitant anterior cruciate ligament reconstruction, classified into early failure (reoperation <365 days, approximating the conventional period of meniscal healing), late failure (≥365 days) or no failure. Cause‐specific Cox proportional hazards regression identified independent predictors of each failure type separately.

**Results:**

Of 38,003 patients, 3399 (8.9%) experienced failure (1996 early; 1403 late). A bimodal hazard pattern supported phenotypic distinction between groups. Early failure was independently predicted by female sex (hazard ratio [HR] 1.15), bucket‐handle tear (HR 1.30), traumatic mechanism (HR 1.14), concomitant microfracture (HR 1.54), hamstring autograft (HR 1.21) and single‐anchor repair (HR 1.28); three or more anchors were protective (HR 0.83). Late failure was uniquely predicted by knee osteoarthritis (HR 1.33), whereas increasing age was independently protective (HR 0.98/year) as well as three or more anchors (HR 0.86). Among isolated meniscal repairs, partial meniscectomy was the most common reoperation in both groups, but more frequent in late failures (60.1% vs. 53.7%). Re‐repair was more common after early failure (22.5% vs. 16.1%), while TKA was more prevalent after late failure (9.8% vs. 5.4%). All reported associations and between‐group differences were statistically significant (*p* < 0.05).

**Conclusion:**

Early and late meniscal repair failures are distinct clinical phenotypes with differing risk factors and reoperation patterns. Early failure appears driven by mechanical and technical factors; late failure is more strongly associated with degenerative joint disease and host biology. These findings may inform individualised monitoring and risk stratification strategies.

**Level of Evidence:**

Level III, retrospective cohort study.

## Graphical abstract

Early versus late meniscal repair failure phenotypes. Take‐home message: Early and late meniscal repair failures demonstrate distinct risk factor profiles, supporting clinically meaningful phenotypes that may guide surveillance and patient counselling.

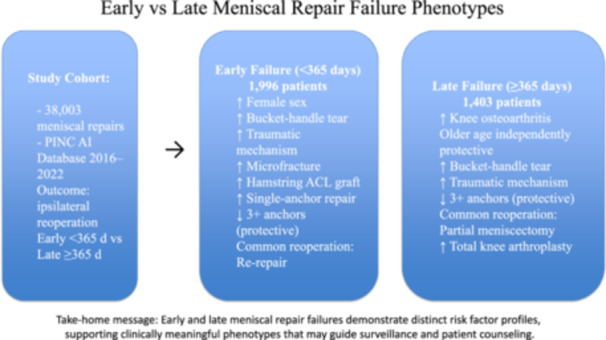

**Abbreviations**
ACL: anterior cruciate ligament
BTB: bone‐patellar tendon‐bone
CI: confidence interval
CPT: current procedural terminology
HR: hazard ratio
ICD‐10: International Classification of Diseases, 10th Revision
LOA/MUA: lysis of adhesions/manipulation under anaesthesia
PINC AI: PINC AI healthcare database
TKA: total knee arthroplasty

## INTRODUCTION

The meniscus is integral to knee joint health, contributing to load transmission, joint stabilisation and articular cartilage protection [11, 43, 45], and bears a majority of the axial load across approximately 60% of the tibiofemoral contact area [2, 27, 44]. Meniscal tissue loss increases tibiofemoral contact pressure and accelerates cartilage degeneration, with complete meniscectomy decreasing contact area by 33%–54% while increasing peak contact pressure by 50%–134%, contributing to the development of osteoarthritis [26, 35]. Consequently, meniscal preservation has become a central priority in the management of meniscal injuries [24, 28, 38].

Reflecting the importance of meniscal preservation, there has been a paradigm shift over the past decades from partial meniscectomy towards meniscal repair in the treatment of meniscal tears, with national trends demonstrating substantial rises in repair rate [1, 3, 19]. Studies have demonstrated that, compared to partial meniscectomy, meniscal repair can improve patient‐reported outcomes and reduce osteoarthritis progression [16, 20, 28]. However, reoperation and failure rates following meniscal repair remain significant concerns, with overall failure rates reported as high as 19.5%–22% [5, 16, 32].

While existing literature has predominantly focused on overall failure rates and associated risk factors, the timing of failure remains poorly characterised, despite clinical observations suggesting that early and late failures may represent mechanistically distinct phenomena, with early failures potentially reflecting mechanical or technical factors related to the repair construct and late failures potentially reflecting degenerative joint disease and host biology [38, 40]. To date, no large‐scale study has directly compared predictors of early versus late meniscal repair failure within the same cohort; existing evidence is limited to indirect signals, such as the association between failed repair and long‐term osteoarthritis progression, and inconsistency in reported risk factors across studies with varying follow‐up durations [37, 41].

Distinguishing between these phenotypes is essential for reducing failure rates and developing targeted prevention strategies. The purpose of this study was to utilise a national database to (1) determine the rates of early (<365 days, approximating the conventional period of meniscal healing) and late (≥365 days) meniscal repair failure, (2) identify predictors differentially associated with each failure cohort and (3) characterise differences in reoperation patterns between the two groups. It was hypothesised that early and late meniscal repair failures would demonstrate distinct predictor profiles consistent with differing underlying biological mechanisms.

## METHODS

### Study design and patient selection

This retrospective cohort study utilised the PINC AI Healthcare Database (formerly Premier Healthcare Database), a large U.S. all‐payer hospital administrative database containing inpatient and hospital‐based outpatient encounters from participating institutions between 2016 and 2022. The database includes patient demographics, diagnosis and procedure codes, and billing data. Because all data were de‐identified, institutional review board approval was not required.

Patients who underwent arthroscopic meniscal repair between 2016 and 2022 were identified using current procedural terminology (CPT) procedure codes. Patients were excluded if they had diagnoses indicating infection, malignancy or had missing key demographic or follow‐up data. Traumatic and non‐traumatic tears, as well as patients younger than 18, were included to reflect the full population undergoing meniscal repair. Each patient was assigned an index procedure date, and analyses were performed at the patient level. Any patient undergoing meniscal repair at the index encounter was eligible for inclusion. Both isolated meniscal repairs and repairs performed with concomitant anterior cruciate ligament (ACL) reconstruction were included, consistent with the intent to capture the full population undergoing meniscal repair. Graft type analysis was restricted to patients undergoing primary ACL reconstruction, patients with evidence of prior ACL reconstruction were excluded from this subanalysis. Procedure and diagnosis code definitions are provided in Supporting Information S1: Table 1.

### Failure classification and follow‐up

The primary outcome was ipsilateral knee reoperation following the index meniscal repair, defined broadly to capture any subsequent ipsilateral knee surgical encounter potentially attributable to the index repair. Reoperations included repeat meniscal repair, partial meniscectomy, meniscal transplantation, knee arthroplasty, synovectomy, chondroplasty, lysis of adhesions, loose body removal, infection‐related procedures and osteotomy procedures. This inclusive definition was chosen because administrative database coding cannot reliably distinguish reoperations performed specifically to address a failed meniscal repair from those performed for other indications at the same surgical encounter; a narrower definition restricted to repeat repair, meniscectomy, transplantation and arthroplasty risks excluding cases in which the index repair contributed to, but was not the sole documented indication for, the reoperation. To ensure that reoperations were attributable to the index knee, laterality modifiers associated with CPT and International Classification of Diseases, 10th Revision (ICD‐10) procedure codes were used. Reoperations without matching laterality to the index procedure were excluded from failure classification to minimise misclassification of contralateral procedures.

**Figure 1:**
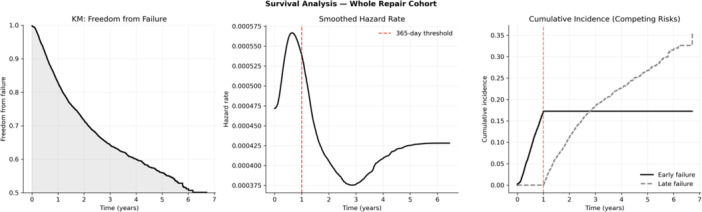
Survival analysis demonstrating bimodal hazard pattern supporting phenotypic distinction between early and late meniscal repair failure. Left: Kaplan–Meier curve depicting cumulative freedom from failure over time. Center: Smoothed hazard rate function with 365‐day threshold (red dashed line); bimodal pattern supports phenotypic distinction between early and late failure groups. Right: Aalen–Johansen cumulative incidence function for early (solid line) and late (dashed line) failure, accounting for competing risks.

Patients were classified into three mutually exclusive groups based on the timing of first reoperation: early failure (reoperation within 365 days), late failure (reoperation at or beyond 365 days), or no failure (no reoperation during follow‐up). The 365‐day threshold was selected to approximate the conventional postoperative period of meniscal biological healing and to align with prior literature reporting failure rates by discrete postoperative year intervals [33]. This threshold was specified a priori as a clinically interpretable cutoff rather than statistically derived from the present dataset, the bimodal hazard distribution subsequently observed in the cohort (Figure 1) provides post hoc support for a meaningful distinction near this time point. Follow‐up time was defined as the number of days from the index procedure to the last recorded hospital encounter. Patients were required to have at least 24 months of follow‐up to be included in the no‐failure group, ensuring adequate observation time to capture both early and late failure events.

### Covariates

Patient‐level covariates included age, sex, insurance status and comorbidities captured using the weighted Van Walraven modification of the Elixhauser comorbidity index and selected individual comorbidities [42]. Tear characteristics were identified using diagnosis codes and included acute versus degenerative tears, medial versus lateral location, bucket‐handle tears and complex tears, characterised by traumatic meniscal injury involving multiple tear patterns or significant tissue disruption. Concomitant ligament injuries and concomitant index procedures were identified using diagnosis and procedure codes. A composite index knee complexity variable was defined as the presence of any concomitant ligament injury or adjunct procedure at the time of index repair. Variables included in multivariable models were selected prior based on clinical relevance.

### Statistical analysis

Baseline characteristics were compared across failure groups using analysis of variance or Kruskal–Wallis tests for continuous variables and chi‐square tests for categorical variables. Time‐to‐event analyses were performed using Kaplan–Meier survival analysis to evaluate freedom from reoperation. Survival time was calculated from the date of index meniscal repair to the date of first reoperation or censoring. Because early and late failure represent competing events, cumulative incidence was estimated using the Aalen–Johansen method for competing risks.

Cause‐specific Cox proportional hazards regression models were used to identify independent predictors of early failure and late failure separately among the full cohort, with concomitant ACL reconstruction included as an adjusted covariate. The proportional hazards assumption was assessed using Schoenfeld residuals. In contrast, reoperation pattern analyses were restricted to patients undergoing isolated meniscal repair without concomitant ligament surgery, because reoperations following concomitant ACL reconstruction may include procedures related to the ligament reconstruction itself (e.g., hardware‐related procedures) rather than the meniscal repair, which could obscure failure‐specific reoperation phenotypes if both groups were combined. Missing data were minimal and were handled using complete‐case analysis. Hazard ratios with 95% confidence intervals (CIs) were reported, and statistical significance was defined as *p* < 0.05. Statistical analyses were performed using Python (version 3.0).

## RESULTS

A total of 38,003 patients undergoing meniscal repair were included (Table 1). Overall, 3399 patients (8.9%) experienced repair failure, with 1996 of the failures within 1 year of repair (defined as early failure) and 1403 after 1 year (defined as late failure). Mean follow‐up was 26.2 months for the early failure group, 28.3 months for the late failure group and 20.4 months for the no‐failure group. Mean age at index repair was 29.7 ± 15.2 years in the early failure group and 28.5 ± 16.1 years in the late failure group, as detailed in Table 1. The early failure group had a higher proportion of female patients than late and no failure groups (52.6% vs. 48.3% vs. 44.1%, *p* < 0.001). Significant differences were observed in tear characteristics, with a higher prevalence of chronic/degenerative tears in the late failure group (8.1%). Additionally, in the no failure group, acute bucket‐handle tears had a prevalence of 12%, while in the early and late failure groups the prevalence was 15.6% and 15.2%, respectively (*p* < 0.001). Concomitant ACL reconstruction was performed in approximately half of all patients, with rates lower in the early failure cohort (47.6%) as compared to the late failure (49.6%) and no failure (51.3%) groups (*p* = 0.003).

**Table 1:** Baseline characteristics by failure timing group.

Variable	No failure (*n* = 34,604)	Early failure (*n* = 1996)	Late failure (*n* = 1403)	*p*‐value
Demographics				
Age (years)	30.3 ± 15.6	29.7 ± 15.2	28.5 ± 16.1	<0.001
Elixhauser comorbidity score	−0.1 ± 1.9	−0.1 ± 1.9	−0.1 ± 1.6	0.171
Female sex	15,263 (44.1%)	1050 (52.6%)	678 (48.3%)	<0.001
Diabetes mellitus	1136 (3.3%)	59 (3.0%)	29 (2.1%)	0.032
Obesity	3509 (10.1%)	190 (9.5%)	104 (7.4%)	0.003
Tobacco use	2547 (7.4%)	127 (6.4%)	78 (5.6%)	0.011
Knee osteoarthritis	1715 (5.0%)	102 (5.1%)	87 (6.2%)	0.109
Medicare	1235 (3.6%)	65 (3.3%)	57 (4.1%)	0.459
Medicaid	6970 (20.1%)	389 (19.5%)	268 (19.1%)	0.509
Private insurance	24,099 (69.6%)	1406 (70.4%)	1004 (71.6%)	0.244
Other/unknown insurance	2300 (6.6%)	136 (6.8%)	74 (5.3%)	0.119
Tear characteristics				
Index knee complexity adjunct	20,029 (57.9%)	1092 (54.7%)	778 (55.5%)	0.005
Acute meniscus tear	31,414 (90.8%)	1820 (91.2%)	1237 (88.2%)	0.003
Chronic/degenerative tear	2142 (6.2%)	119 (6.0%)	114 (8.1%)	0.012
Acute medial tear	14,048 (40.6%)	851 (42.6%)	551 (39.3%)	0.111
Acute lateral tear	2133 (6.2%)	111 (5.6%)	73 (5.2%)	0.199
Chronic medial tear	914 (2.6%)	52 (2.6%)	50 (3.6%)	0.108
Chronic lateral tear	302 (0.9%)	18 (0.9%)	18 (1.3%)	0.276
Bucket‐handle tear (acute)	4140 (12.0%)	311 (15.6%)	213 (15.2%)	<0.001
Complex tear (acute)	13,398 (38.7%)	708 (35.5%)	468 (33.4%)	<0.001
Loose body	1432 (4.1%)	77 (3.9%)	51 (3.6%)	0.550
Traumatic mechanism	18,825 (54.4%)	1053 (52.8%)	715 (51.0%)	0.017
ACL injury	17,589 (50.8%)	968 (48.5%)	675 (48.1%)	0.021
Chondromalacia/cartilage disorder	4524 (13.1%)	254 (12.7%)	183 (13.0%)	0.904
Concomitant procedures				
Concomitant ACL reconstruction	17,756 (51.3%)	950 (47.6%)	696 (49.6%)	0.003
Capturable grafts^a^	11,008 (62.0%)	555 (58.4%)	411 (59.1%)	0.030
BTB graft^b^	6054 (54.7%)	324(52.1%)	227 (55.3%)	0.295
Hamstring graft^b^	4954 (45.3%)	231 (47.9%)	184 (44.7%)	0.295
Both menisci involved	4662 (13.5%)	318 (15.9%)	204 (14.5%)	0.005
Concomitant microfracture	1,771 (5.1%)	118 (5.9%)	67 (4.8%)	0.241
Anchor use				
1	9703 (28.0%)	590 (29.6%)	355 (25.3%)	0.003
2	7290 (21.1%)	451 (22.6%)	257 (18.3%)	0.010
3+	8329 (24.1%)	511 (25.6%)	299 (21.3%)	0.020
Not captured	9283 (26.8%)	444 (22.2%)	492 (35.0%)	<0.001

*Note*: Early failure defined as reoperation within 365 days of index procedure; late failure defined as reoperation ≥365 days after index procedure. A composite index knee complexity adjunct variable was defined as the presence of any concomitant ligament injury or adjunct procedure at the time of index repair. *p*‐values reflect chi‐square tests for categorical variables and one‐way analysis of variance for continuous variables.

Abbreviations: ACL, anterior cruciate ligament; BTB, bone‐patellar tendon‐bone.

a Denominator = patients with concomitant ACL reconstruction in each group.

^b^ Denominator = capturable grafts in each group.

Kaplan–Meier freedom from failure and smoothed hazard rate analyses demonstrated a bimodal failure pattern supporting phenotypic distinction between early and late failure (Figure 1). An Aalen–Johansen cumulative incidence function further illustrated divergent trajectories between early and late failure groups: cumulative incidence of early failure rose sharply within the first postoperative year before plateauing, by definition, after the 365‐day threshold, whereas cumulative incidence of late failure remained negligible during the first year before rising steadily and continuing to accrue throughout the remainder of follow‐up (Figure 1, right panel).

Cause‐specific Cox regression analysis revealed distinct predictor profiles for early and late failure phenotypes (Table 2). Female sex was independently predictive of early failure (hazard ratio [HR]: 1.15, 95% CI: 1.05–1.26). Concomitant microfracture was also significantly predictive of early failure alone (HR: 1.54, 95% CI: 1.08–2.18), as was using hamstring autograft in concomitant ACL reconstructions (HR: 1.21, 95% CI: 1.04–1.38). BTB reconstruction was found to be protective against early failure (HR: 0.84, 95% CI: 0.78–0.90). In contrast, late failure was uniquely predicted by knee osteoarthritis (HR: 1.33, 95% CI: 1.04–1.69). Bucket‐handle tears predicted both early (HR: 1.30, 95% CI: 1.12–1.52) and late (HR: 1.32, 95% CI: 1.10–1.59) failure. Traumatic injury mechanism was also predictive of early (HR: 1.14, 95% CI: 1.04–1.25) and late failure (HR: 1.15, 95% CI: 1.02–1.28). The use of three or more anchors was protective against both early (HR: 0.83, 95% CI: 0.79–0.86) and late failure (HR: 0.86, 95% CI: 0.80–0.92), while the use of a single anchor was predictive of early failure (HR: 1.28, 95% CI: 1.06–1.55). Older age was independently protective against both early (HR: 0.99 per year, 95% CI: 0.99–0.99) and late failure (HR: 0.98 per year, 95% CI: 0.97–0.98), indicating a small but significant decrease in failure hazard with each additional year of age. Medicaid payer status was independently protective in early (HR: 0.79, 95% CI: 0.70–0.88) and late failure cohorts (HR: 0.69, 95% CI: 0.60–0.79). Medicare was found to be protective against early and late failure (HR: 0.76, 95% CI: 0.58–0.99, HR: 0.69, 95% CI: 0.60–0.79).

**Table 2:** Cause‐specific cox proportional hazards regression: Independent predictors of early and late meniscal repair failure.

Variable	Early HR (95% CI)	Early *p*‐value	Late HR (95% CI)	Late *p*‐value
Age	0.99 (0.99–0.99)	<0.001	0.98 (0.97–0.98)	<0.001
Female sex	1.15 (1.05–1.26)	0.003	0.93 (0.83–1.03)	0.164
Elixhauser score	1.01 (0.98–1.04)	0.521	0.97 (0.94–1.01)	0.139
Diabetes mellitus	0.93 (0.70–1.22)	0.596	0.67 (0.45–0.98)	0.041
Obesity	1.00 (0.83–1.21)	0.977	0.76 (0.59–0.98)	0.038
Tobacco use	0.95 (0.79–1.14)	0.576	0.98 (0.77–1.24)	0.841
Knee osteoarthritis	0.95 (0.77–1.18)	0.634	1.33 (1.04–1.69)	0.021
Medicare	0.76 (0.58–0.99)	0.041	1.02 (0.76–1.37)	0.885
Medicaid	0.79 (0.70–0.88)	<0.001	0.69 (0.60–0.79)	<0.001
Acute meniscus tear	1.08 (0.82–1.42)	0.577	0.93 (0.68–1.27)	0.650
Chronic meniscus tear	0.99 (0.71–1.40)	0.974	0.89 (0.61–1.30)	0.539
Acute medial meniscus tear	1.02 (0.90–1.16)	0.741	0.98 (0.84–1.14)	0.792
Acute lateral meniscus tear	0.97 (0.78–1.20)	0.764	0.97 (0.75–1.26)	0.846
Bucket‐handle tear (acute)	1.30 (1.12–1.52)	0.001	1.32 (1.10–1.59)	0.003
Complex tear (acute)	0.94 (0.83–1.06)	0.301	0.94 (0.81–1.10)	0.443
Chronic medial meniscus tear	0.99 (0.68–1.44)	0.950	1.05 (0.72–1.55)	0.788
Chronic lateral meniscus tear	1.12 (0.67–1.88)	0.660	1.01 (0.60–1.70)	0.956
ACL injury	1.17 (0.95–1.45)	0.143	0.80 (0.63–1.03)	0.089
Chondromalacia	0.91 (0.79–1.04)	0.157	1.04 (0.88–1.22)	0.674
Loose body	1.05 (0.82–1.36)	0.682	0.95 (0.69–1.31)	0.763
Traumatic mechanism	1.14 (1.04–1.25)	0.007	1.15 (1.02–1.28)	0.018
Concomitant ACL reconstruction	0.90 (0.60–1.35)	0.617	1.18 (0.68–2.05)	0.547
BTB Recon	0.84 (0.78–0.90)	<0.001	1.04 (0.86–1.22)	0.258
Hamstring	1.21 (1.04–1.38)	0.043	1.32 (0.86–1.78)	0.184
Concomitant microfracture	1.54 (1.08–2.18)	0.017	1.19 (0.73–1.95)	0.488
Index knee complexity adjunct	0.81 (0.56–1.18)	0.267	1.00 (0.59–1.68)	0.988
1+	1.28 (1.06–1.55)	0.011	0.86 (0.71–1.03)	0.101
2+	1.02 (0.96–1.08)	0.512	1.04 (0.99–1.10)	0.158
3+	0.83 (0.79–0.86)	<0.001	0.86 (0.80–0.92)	<0.001

*Note*: Early failure defined as reoperation <365 days; late failure defined as reoperation ≥365 days after index procedure. All models adjusted for variables listed. Anchor use reference category = no anchor captured.

Abbreviations: ACL, anterior cruciate ligament; BTB, bone‐patellar tendon‐bone; CI, confidence interval; HR, hazard ratio.

**Figure 2:**
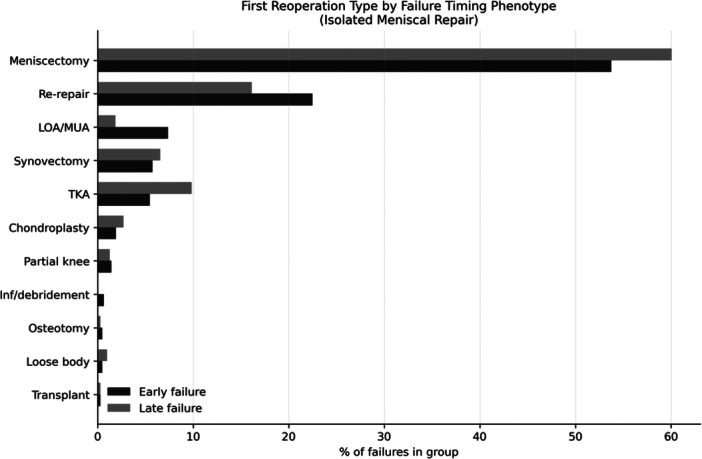
First reoperation type by failure timing phenotype among isolated meniscal repair failures. Horizontal bars represent the percentage of patients within each failure group undergoing each reoperation type as their first subsequent procedure. Restricted to patients undergoing isolated meniscal repair. Black bars = early failure (<365 days); grey bars = late failure (≥365 days). LOA/MUA, lysis of adhesions/manipulation under anaesthesia; TKA, total knee arthroplasty.

Reoperation patterns differed between the early and late failure groups among patients undergoing isolated meniscal repair (Figure 2, Table 3). Partial meniscectomy was the most common reoperation in both groups, comprising a significantly larger proportion of late failure than early failure reoperations (60.1% vs. 53.7%, *p* = 0.009). Re‐repair was the second most common early failure reoperation (22.5% vs. 16.1%, *p* = 0.001), while lysis of adhesions/manipulation under anaesthesia (LOA/MUA) was more frequent in early failures (7.4% vs. 1.9%, *p* < 0.001). Total knee arthroplasty (TKA) was more prevalent among late failures (9.8% vs. 5.4%, *p* < 0.001). Together, partial meniscectomy and re‐repair accounted for 76.2% of early failure reoperations and 76.2% of late failure reoperations, with the remaining reoperation types (chondroplasty, synovectomy, lysis of adhesions/manipulation under anaesthesia, loose body removal, osteotomy, infection‐related procedures and transplantation) collectively comprising less than one‐quarter of reoperations in each group.

**Table 3:** First reoperation type among isolated meniscal repair failures stratified by failure timing phenotype.

Reoperation type	Early failure *N* (%)	Late failure *N* (%)	*p*‐value
Chondroplasty	20 (1.9%)	19 (2.7%)	0.238
Infection/debridement	7 (0.7%)	0 (0.0%)	0.089
LOA/MUA	77 (7.4%)	13 (1.9%)	<0.001
Loose body	5 (0.5%)	7 (1.0%)	0.276
Partial meniscectomy	562 (53.7%)	421 (60.1%)	0.009
Osteotomy	5 (0.5%)	2 (0.3%)	0.726
Partial knee	15 (1.4%)	9 (1.3%)	0.841
Re‐repair	235 (22.5%)	113 (16.1%)	0.001
Synovectomy	60 (5.7%)	46 (6.6%)	0.438
TKA	57 (5.4%)	69 (9.8%)	<0.001
Transplant	3 (0.3%)	2 (0.3%)	1.000
Total	1046	701	

*Note*: Restricted to patients undergoing isolated meniscal repair without concomitant procedures. Early failure defined as reoperation <365 days; late failure defined as reoperation ≥365 days after index procedure. *p*‐values reflect chi‐square or Fisher's exact tests as appropriate.

Abbreviations: LOA/MUA, lysis of adhesions/manipulation under anaesthesia; TKA, total knee arthroplasty.

## DISCUSSION

The purpose of this study was to characterise early and late meniscal repair failure as potentially distinct clinical phenotypes by comparing their incidence, independent predictors and reoperation patterns within a large national cohort. Consistent with the hypothesis, early and late failure demonstrated distinct predictor profiles: early failure was predominantly associated with repair construct and technical factors, including single‐anchor fixation, concomitant microfracture and hamstring autograft use, while late failure was more strongly associated with host and degenerative factors, including knee osteoarthritis and older age. The results of this study suggest that early and late meniscal repair failure may represent distinct clinical phenotypes with differing risk factor profiles, rather than a single biological process occurring uniformly over time. The observed bimodal hazard pattern is consistent with this interpretation, though it does not itself confirm that failures arising near the threshold represent fundamentally different biological processes. Nevertheless, the divergent predictor profiles identified for each phenotype support clinically meaningful differences in the factors associated with early versus late failure, which may inform patient counselling, surgical technique, and postoperative surveillance strategies [40, 47].

Prior studies evaluating meniscal repair failure have generally reported overall failure rates and risk factors without differentiating timing of failure [7, 32, 41]. Reported risk factors include age, tear pattern and concomitant procedures [18, 40, 43], but these studies typically evaluated failure as a single outcome, which may explain inconsistent findings across the literature. The present study suggests that risk factors differ substantially between early and late failure, which may reconcile previously conflicting results.

Early failure was predominantly associated with repair construct, surgical technique and early postoperative joint environment, suggesting a mechanical and technical mechanism. Female sex emerged as an independent predictor of early failure but was not associated with late failure, supporting the concept that early failure may be influenced by biologic healing characteristics rather than long‐term tissue durability. A few proposed explanations include differences in ligamentous laxity, hormonal influences on collagen metabolism and anatomic differences affecting meniscal loading and healing environment [14, 15]. Hewett et al. demonstrated that fluctuating levels of oestrogen, progesterone and relaxin across the menstrual cycle are associated with increased ligamentous laxity and reduced neuromuscular performance, which may compromise knee stability during the early postoperative healing period and place greater mechanical stress on a healing meniscal repair [15]. Additionally, Helito et al. identified female sex as an independent risk factor for medial meniscus posterior root repair failure, proposing that sex‐related differences in coronal plane alignment and associated meniscal loading may disrupt the biological healing environment following repair [14]. Together, these mechanisms suggest that the influence of female sex on early meniscal repair failure may be driven by a combination of hormonally mediated tissue properties and biomechanical loading patterns rather than intrinsic tissue durability over time.

Older age was independently protective against both early and late failure, which may appear counterintuitive but likely reflects surgical selection bias, as older patients undergoing meniscal repair may represent a subset in whom surgeons have deemed tear characteristics and tissue quality favourable for healing. Unlike female sex, which was selectively associated with early failure only, the protective effect of age was consistent across both phenotypes, suggesting these variables influence repair outcomes through distinct pathways.

Bucket‐handle tears were associated with increased risk of both early and late failure, suggesting tear severity, size and higher energy trauma is a risk factor independent of failure phenotype. This is consistent with prior literature showing that bucket‐handle tears carry substantially higher failure rates than simpler tear patterns, with reported rates ranging from 19% to 34% across multiple studies [23, 29, 30]. This is notably higher than the 15.2%–15.6% observed in the present study, which may reflect differences in failure ascertainment and cohort composition rather than the inclusiveness of the reoperation‐based definition. Muench et al. defined failure using clinical and radiographic healing criteria, including persistent symptoms and second‐look or imaging‐based evidence of incomplete healing, which can identify failure in patients managed conservatively who never undergo a subsequent surgical procedure and would therefore not be captured by a reoperation‐based definition such as this [30]. Kekki et al. similarly used a reoperation‐based failure definition but restricted their cohort to displaced bucket‐handle tears, a more severe and unstable tear subtype than the broader acute bucket‐handle category captured in the present study, which may explain their higher observed failure rate [23]. These comparisons highlight that differences in failure ascertainment method and tear severity, rather than the breadth of reoperation types included in a given definition, are likely the primary drivers of variability in reported bucket‐handle failure rates across studies [6, 23, 29, 30, 34]. A similar pattern was observed for involvement of both menisci at the index procedure, which was more common among early failures than late or no‐failure groups (15.9% vs. 14.5% vs. 13.5%, respectively; Table 1). As with bucket‐handle tears, this may reflect a greater burden of traumatic mechanism or more extensive joint injury at the time of index surgery, though this variable was not independently significant in multivariable modelling and should be interpreted as a univariable association only.

Concomitant microfracture was strongly associated with early failure, which warrants careful interpretation. The mean age of patients undergoing concomitant microfracture was 30.5 years, comparable to the overall cohort mean, suggesting that age‐related chondral degeneration alone does not fully explain the indication for microfracture in this population. In the context of isolated meniscal repair without concomitant ACL reconstruction, microfracture is likely being done as a biological augmentation to deliver bone marrow contents to the repair site and promote healing rather than to address chondral pathology [13, 22]. Notably, this finding contrasts with prior literature on intentional marrow‐venting or microfracture augmentation of isolated meniscal repair, which has generally reported comparable or improved healing outcomes relative to repair without augmentation. Dean et al. found no significant difference in failure rates between isolated meniscal repair augmented with a marrow venting procedure and meniscal repair performed with concomitant ACL reconstruction (12.9% vs. 7.8%, *p* = 0.429), and other comparative studies of intercondylar notch microfracture augmentation have similarly reported improved or comparable healing relative to non‐augmented repair [8, 22]. This discrepancy may reflect a fundamental difference between studies of intentional, protocolized augmentation performed for biological enhancement and the present study's administrative database approach, in which concomitant microfracture coding may instead serve as a proxy for unmeasured chondral pathology, tear severity or other factors associated with a more complex index procedure, rather than capturing a true causal effect of the augmentation technique itself. Furthermore, as microfracture was not stratified by concomitant ACL reconstruction status in the analysis, it is possible that the observed association reflects differing indications across surgical contexts that is unable to be fully disentangled with the available data. The association with early failure may reflect the limitations of administrative coding or differences in surgical indication rather than a harmful effect of microfracture itself. The search for an effective biological augmentation strategy in isolated meniscus repair is far from over, and future investigation into techniques such as fibrin clots, platelet‐rich plasma and other orthobiologics is warranted.

Repair construct characteristics also appeared important in early failure risk. Single‐anchor repairs were associated with increased early failure, while repairs using three or more anchors were protective, likely reflecting construct stability and load distribution across the healing meniscus. Biomechanical studies support this principle, demonstrating that increasing the number of fixation points improves load‐to‐failure strength and reduces gapping at the repair site under cyclic loading conditions [4, 17, 37]. However, anchor count may serve as a proxy for tear length and complexity and relies on variable coding accuracy, so this finding should be interpreted cautiously.

Among patients undergoing concomitant ACL reconstruction, BTB autografts were protective against early meniscal repair failure while hamstring autografts were associated with increased risk, a finding consistent with the broader ACL reconstruction literature [21, 39]. Large registry studies and meta‐analyses have consistently demonstrated lower ACL revision rates with BTB compared to hamstring autografts. A meta‐analysis of over 47,000 patients found a significantly lower graft rupture rate with BTB autograft [39], and a registry study of young athletes demonstrated that hamstring autograft recipients had more than twice the risk of ACL revision compared to BTB recipients [21]. The superior rotational stability, earlier osseointegration through bone‐to‐bone healing, and greater initial construct stiffness associated with BTB grafts likely produce a more mechanically stable postoperative knee environment, which may be protective of the healing meniscus during the early postoperative period [10, 12]. That graft type independently predicted early meniscal repair failure after adjusting for other covariates is an intriguing finding, though its interpretation is limited by several factors not captured in the database, including age differences between graft type groups, associated LET/ALL augmentation procedures, meniscal tear laterality and postoperative rehabilitation protocols. These unmeasured variables may confound the observed association, and the finding should therefore be interpreted as hypothesis‐generating rather than definitive evidence that graft selection influences meniscal repair outcomes.

The reoperation distribution further supports the mechanical nature of early failure. Among patients undergoing isolated meniscal repair, re‐repair was the second most common early failure reoperation (22.5%), though re‐repair rates may also reflect patient age, as younger patients are more likely to be offered re‐repair regardless of failure mechanism, and age was not accounted for in the reoperation pattern analysis. Lysis of adhesions and manipulation under anaesthesia were more common in early failures (7.4% vs. 1.9%, *p* < 0.001), though rates were substantially lower than in the full cohort, suggesting that much of the LOA/MUA burden in the broader population is driven by concomitant ACL reconstruction rather than meniscal repair failure itself. The residual LOA/MUA rate in this isolated repair cohort is nonetheless higher than expected. Prior database studies have reported MUA and/or LOA rates of approximately 0.5% within 3–6 months following isolated meniscal repair, and isolated meniscal procedures have consistently been associated with the lowest rates of postoperative MUA/LOA among arthroscopic knee procedures, in contrast to the substantially higher rate observed among early failures in the present cohort [36, 46]. This discrepancy may represent a coding artefact inherent to administrative database research, such as miscapture of concomitant minor procedures performed at the time of a subsequent reoperation.

In contrast, late failure was more strongly associated with host factors and joint degeneration. Older age, osteoarthritis and degenerative tear patterns were preferentially associated with late failure, supporting the thinking that late failure represents progression of joint disease rather than isolated repair failure [31, 38, 43]. Partial meniscectomy was the most common late reoperation, suggesting that by the time late failure occurs, surgeons believe remaining meniscal tissue is often biologically no longer suitable for re‐repair. Progression to TKA was also more frequent in late failures, further supporting the interpretation that late failure is more closely tied to osteoarthritis progression than repair construct failure.

An unexpected finding was that Medicaid insurance status was associated with decreased risk of both early and late failure. This is counterintuitive given that Medicaid populations often face barriers to care and worse outcomes in orthopaedic conditions [9, 25]. Possible explanations include lower postoperative activity levels, younger patient age within this cohort or surveillance bias. Medicaid patients may be less likely to return to the same hospital system for reoperation despite symptomatic failure. This finding should not be interpreted as evidence of superior outcomes in Medicaid populations, but rather as a prompt for further investigation into how follow‐up patterns and access‐to‐care differences affect administrative database studies.

This study has several limitations inherent to administrative database research. The PINC AI database lacks detailed clinical information such as imaging findings, specific intraoperative tear characteristics, rehabilitation protocols and patient‐reported outcomes. Failure was defined by reoperation, which likely underestimates true failure rates. Additionally, the failure definition included reoperation types such as chondroplasty, synovectomy, lysis of adhesions, loose body removal and osteotomy, which may not in all cases represent a true failure of the meniscal repair itself. This broad definition was adopted to minimise undercounting of reoperations attributable to the index repair given the limitations of administrative coding, but it is possible that some included procedures were performed for indications unrelated to repair failure, which could bias failure rates and associated predictors toward the null. Although laterality modifiers were used to attribute reoperations to the index knee, misclassification remains possible when laterality coding is inconsistent. Additionally, while medial versus lateral tear location and concomitant ACL reconstruction status were included as covariates in the regression models, this study was not powered or designed to evaluate stratified or interaction effects between tear location, tear pattern (e.g., medial vs. lateral bucket‐handle tears), and ACL status; such granular subgroup analyses represent an important direction for future investigation. Follow‐up was limited to encounters within participating hospitals, introducing potential non‐random loss to follow‐up. Additionally, the database's 2016–2022 date range limited maximum follow‐up to approximately 6 years, precluding subgroup analysis of late failure by longer‐term intervals. Surgical variables such as anchor count, graft type and tear morphology were derived from billing codes and represent proxies rather than direct operative data, and important factors including surgeon experience, rehabilitation protocols, activity level and patient compliance were not captured. Also, administrative coding does not allow identification of specific tear subtypes such as ramp or root lesions, which may be grouped within broader tear classifications and carry distinct prognoses. Detailed laterality information at the tear level was also unavailable, precluding stratified analysis by medial versus lateral repair.

These findings carry direct clinical relevance for the management of patients undergoing meniscal repair. Recognising early and late failure as phenotypically distinct entities, rather than a single outcome, may allow surgeons to tailor postoperative monitoring strategies based on individual risk profiles. Patients with technical risk factors such as limited fixation constructs or soft‐tissue ACL grafts may warrant closer early surveillance, while those with degenerative features or osteoarthritis may benefit from longer‐term follow‐up. Prospective validation of these phenotypes remains an important next step.

## CONCLUSION

Early and late meniscal repair failure demonstrate distinct risk factor profiles, suggesting they may represent clinically meaningful phenotypes rather than a single uniform outcome. Early failure was associated with technical and construct‐related factors, while late failure was more strongly associated with degenerative joint disease and host biology, though these findings reflect statistical associations rather than directly demonstrated biological or technical mechanisms. Patients with complex tears, limited fixation constructs, without concomitant ligamentous surgery, or with use of soft tissue ACL graft types may benefit from closer early postoperative monitoring, whereas those with degenerative tears, osteoarthritis or older age may require longer‐term surveillance for degenerative progression. Future research should prospectively validate these phenotypes using imaging, specific tear characteristics, patient‐reported outcomes and detailed surgical technique.

## AUTHOR CONTRIBUTIONS

All authors contributed to the conception and design of the study, acquisition and interpretation of data, drafting and critical revision of the manuscript for important intellectual content, final approval of the version to be published, and agree to be accountable for all aspects of the work. Jacob W. McDevitt had full access to all the data in the study and takes responsibility for the integrity of the data and the accuracy of the data analysis. IRB approval was not sought for this study as all data is de‐identified.

## FUNDING INFORMATION

The authors have no funding to report.

## CONFLICT OF INTEREST STATEMENT

Vehniah K. Tjong is a consultant for Smith & Nephew. The remaining authors declare no conflicts of interest.

## ETHICS STATEMENT

IRB approval was not sought for this study as all data are de‐identified.

## Supporting information

Supporting File 1.

## Data Availability

The data underlying this study were obtained from the PINC AI Healthcare Database under a data use agreement and are not publicly available. Analytic code is available from the corresponding author upon reasonable request.
