# Supplementary material for: Not all meniscal repair failures are equal: A comparison between early and late failure risk factors
Source: J Exp Orthop. 2026 Jul 20;13(3):e70856. doi: 10.1002/jeo2.70856 (PMC13384345; doi:10.1002/jeo2.70856)
Supplement: Supplementary file 1 — Supporting File 1. [file JEO2-13-e70856-s001.docx]

Supplemental Table 1: CPT and ICD Codes Utilized

| **Category** | **Code Type** | **Codes** |
| --- | --- | --- |
| Index Procedure (Inclusion) | CPT | 29882, 29883 |
| Concomitant Index Procedures | CPT | 29888, 27407, 27409, 27405, 29889, 27412, 27415, 29866, 29867, 27416, 29870, 29879, 29877, 29874, 29884, 27570, 29875, 29876, 27425, 29873, 27418, 27420, 27422, 27424, 27457, 27450, 27455, 29894 |
| Reoperations | CPT | 29881, 29880, 29882, 29883, 29868, 27447, 27446, 29875, 29876, 29877, 29884, 27570, 29874, 27301, 27303, 27310, 29871, 27457, 27450 |
| Tear Morphology/Location | ICD-10-CM | S83.2, M23.2, S83.24, S83.27, M23.22, M23.26, S83.21, S83.26, S83.23, S83.28 |
| Ligament Injuries | ICD-10-CM | S83.51, S83.52, S83.41, S83.42 |
| Associated Diagnoses | ICD-10-CM | M17, M94, M23.4, M23.3, V, W, X, Y |
